# Synthesis of isocyanate containing cryogels for fast (bio)molecular immobilization

**DOI:** 10.55730/1300-0527.3696

**Published:** 2024-10-08

**Authors:** Merve BAT ÖZMATARA, Tuğçe Nihal GEVREK

**Affiliations:** Department of Chemistry, Gebze Technical University, Gebze, Kocaeli, Turkiye

**Keywords:** Reactive polymers, cryogels, bioimmobilization, isocyanate-amine

## Abstract

Cryogels containing isocyanate reactive groups were synthesized via photopolymerization of 2-isocyanoethyl methacrylate (ICEMA) and poly(ethylene glycol) methyl ether methacrylate (PEGMEMA). By changing the PEGMEMA and ICEMA ratios, cryogel series with varying ratios of reactive isocyanate groups were successfully prepared. The cryogels were characterized using Fourier transform infrared spectroscopy (FTIR), thermogravimetric analysis, scanning electron microscope, and rheometry. To demonstrate that molecules containing amine groups can be immobilized onto synthesized cryogels through isocyanate–amine reactions, the cryogels were conjugated with 4-(trifluoromethyl)benzylamine (TFBA) and fluorescein amine (FLA) molecules, and the conjugations were confirmed through FTIR and fluorescence microscopy, respectively, for TFBA and FLA. Additionally, immobilization of fluorescein isothiocyanate conjugated albumin from bovine serum (FITC-BSA) as fluorescein-labeled model proteins was studied to illustrate that biomolecules can also be bound to the cryogels without any linker. It was shown that the amount of immobilized FITC-labeled model proteins can be controlled by adjusting the concentration of isocyanate reactive groups within the cryogel matrix, and this functionalization was confirmed by fluorescence microscope.

## 1. Introduction

Cryogels are spongy and elastic gels with high surface area and absorption capacity, formed by cryogelation at subzero temperatures (typically between −5 and −20 °C) [1]. Cryogels can be synthesized with specific physical, chemical, mechanical, and biological properties [2] for various fields such as biosensors, biocatalyst immobilization, tissue engineering, and drug transport [3–5]. The highly porous structure of cryogels and their spongy and elastic properties provide various advantages in biomolecule immobilization with reactive functional groups, allowing these structures to be used in biomedical fields such as separation and diagnosis of specific proteins [6–9]. Due to its ability to perform efficient chemical transformations under mild conditions, there has been great interest in using ‘click’ chemistry tools in the functionalization of polymeric materials in recent years [10–14]. Since harsh conditions may cause denaturation of biomolecules, it is desirable to carry out immobilization in mild conditions and preferably without a catalyst [15–17]. For this reason, the maleimide functional group was used in immobilization due to its high reactivity to thiol groups under mild conditions [18,19]. UV-initiated radical thiol-ene click chemistry, which does not require harsh reaction conditions and does not contain by-products, is also widely used in both polymer synthesis and modification [20,21].

Alternatively, the isocyanate group can be utilized for fast and efficient functionalization of polymeric materials. It reacts fast and effectively with amine-containing molecules without the need for any additional catalyst [22]. Isocyanatoethyl(meth)acrylate monomers have been utilized to prepare amine or thiol reactive polymers [23–25] and in the fabrication of polymer brushes [26]. Probably due to concerns about the high reactivity of this monomer there are fewer examples of reactive polymers with the isocyanate unit in the literature when compared with other commercial amine-reactive monomers such as glycidyl methacrylate [27–31], aldehydes [32–35], or the highly studied amine reactive monomer N-hydroxy succinimide [36–40]. Actually, handling of this monomer is not easy but not as hard as one may presume. When polymerization is carried out in an organic solvent followed by prompt functionalization with the molecule/biomolecule of interest, the synthesis of polymers containing this group along with hydrophilic comonomers is possible. Our research group reported the synthesis of PEG-based isocyanate containing 2D and 3D hydrogels and successful immobilization of thiol and amine containing small molecules and protein-binding ligands using organic solvents [41]. After attachment of a ligand in organic solvent, ligand-directed protein immobilization was performed in aqueous medium. In a previous paper, we reported the preparation of sulfobetaine-based isocyanate containing hydrogel films on a glass surface and showed direct immobilization of amine-containing proteins and enzymes on those films [42]. The findings from these studies demonstrated that functionalizing gels through isocyanate–amine reactions is quite straightforward. This led us to consider that three-dimensional polymeric materials containing isocyanate could be prepared with a porous structure, which would offer a larger surface area, thereby providing a more advantageous platform for effective bioimmobilization.

Herein we report the synthesis of free isocyanate containing cryogels for the first time and demonstrate direct attachment of amine-containing (bio)molecules to those cryogels. The crosslinking reaction took place in an organic solvent within a short time, allowing the isocyanate monomer to polymerize with poly(ethylene glycol) methyl ether methacrylate (PEGMEMA) without the decomposition of isocyanate units. Notably, functionalization via the amine–isocyanate reaction was also demonstrated for the first time on cryogel structures, and successful conjugation was confirmed through Fourier transform infrared spectroscopy (FTIR) analysis and fluorescence microscopy. To demonstrate the applicability of these structures in bio-based applications, bioimmobilization experiments were conducted using fluorescein isothiocyanate conjugated albumin from bovine serum (FITC-BSA). The results obtained show that those materials are quite promising for bioimmobilization-based research.

## 2. Materials and methods

### 2.1. Materials and instrumentation

2-Isocyanatoethyl methacrylate (ICEMA) and 2,2-dimethoxy-2-phenylacetophenone (DMPA) were purchased from Chem Cruz. 3-Amino-1-propanol (3-AP), poly(ethylene glycol) methyl ether methacrylate (PEGMEMA, Mn: 300), poly(ethylene glycol) diacrylate (PEGDA, Mn: 575), and fluoresceinamine isomer-I (FLA) were purchased from Sigma Aldrich. Tetrahydrofuran (THF) was purchased from Merck. 1,4-Dioxane and albumin from bovine serum (BSA)-FITC conjugate were purchased from Fisher Scientific. 4-(Trifluoromethyl)benzylamine (TFBA) was purchased from Chem Cruz.

Photopolymerization reactions were performed at 365 nm using a UV lamp (100 W, Analytikjena). An attenuated total reflection (ATR)-Fourier transform infrared (FTIR) spectrometer (PerkinElmer Paragon 100 ATR-IR) was used for chemical characterization of cryogels. The morphology of the cryogels was observed using a JEOL Neoscope JCM-5000 scanning electron microscope. The rheological behavior of the cryogels was evaluated by measuring the loss (G″), storage (G′) moduli, and frequency sweep test of the cryogel as a function of strain using an Anton Paar MCR 302 rheometer. Thermal analysis of the gels was performed under N_2_ gas flow with a Mettler Toledo model TGA/851. Fluorescence microscopy images were recorded at room temperature on a Zeiss Observer.Z1 fluorescent microscope (ZEISS Fluorescence Microscopy, Carl Zeiss Canada Ltd, Canada).

### 2.2. Experimental methods

#### 2.2.1. Fabrication of cryogels

Cryogels were synthesized by photopolymerization using ICEMA, PEGMEMA with PEGDA crosslinker, and DMPA photoinitiator. Using the ICEMA:PEGMEMA ratios 10:40, 25:25, and 40:10 and keeping the crosslinker, initiator, and solvent amounts constant, C1–C3 cryogels were synthesized. A representative procedure for C1 is as follows: a mixture of ICEMA (20.17 mg, 0.13 mmol), PEGMEMA (156 mg, 0.52 mmol), PEGDA (373 mg 0.65 mmol), and DMPA (3.34 mg, 0.0130 mmol) were prepared in 1,4-dioxane (1.5 mL) and kept in a syringe at −70 °C overnight, and then kept under UV irradiation (365 nm, 100 W) for 1.5 h at −13 °C. After the gelation reaction, the cryogel was washed several times with 1,4-dioxane followed by THF to remove all unreacted reactants and dried in vacuo.

#### 2.2.2. Water uptake study of cryogels

Swelling capacities of the synthesized cryogels were determined by keeping the dried cryogel in a beaker containing distilled water at room temperature. At periodic time points, the increase in the mass of the cryogel sample was recorded after the cryogel was removed from the water and the excess water adhered to the surface was absorbed with tissue paper. The percent water uptake was obtained using the equation


(1) $$\text{Water uptake }(\%)=({\text{W}}_{\text{wet}}-{\text{W}}_{\text{dry}})/{\text{W}}_{\text{dry}}\times 100,$$

where W_wet_ and W_dry_ are the weights of the cryogels in the swollen and dried states, respectively.

The porous structure of the cryogels was investigated using scanning electron microscope (SEM) measurements. The average diameter (D) of the pores was calculated using the image processing software ImageJ with data from 100 pores consisting of three SEM images at 250× magnification.

#### 2.2.3. Measurements of deswelling and reswelling kinetics

For deswelling kinetics measurements, the water-swelled cryogel was transferred to acetone. The volume change of the gels was determined by measuring the diameter of the cryogel at regular time intervals. To measure the reswelling kinetics of the cryogels, gel samples that collapsed at equilibrium were transferred back into water. Volume changes of gels were determined using the following formula [43]:


(2) $$Vrel={\left(\frac{Dt}{D}\right)}^{3},$$

where Dt is the diameter of the gel sample at time t and D is its equilibrium swollen diameter in water.

#### 2.2.4. Functionalization of cryogels with amine containing molecules (TFBA)

The amount of TFBA was calculated according to the amount of isocyanate in the cryogel that is used for the synthesis assuming 100% conversion; 20 mg of C1 cryogel contains 0.004734 mmol isocyanate, C2 cryogel contains 0.01247 mmol isocyanate, and C3 cryogel contains 0.0211 mmol isocyanate. Each cryogel (20 mg) was kept in TFBA solution (prepared with THF) containing the same number of moles of isocyanate as it contained, for 30 min. The cryogels were then washed several times with THF and dried in vacuo. As a control gel, 20 mg of C1 cryogel was kept in a solution prepared in 0.047 mmol (3.53 mg) 3-AP and 20 μL of THF for 30 min and washed with THF. This cryogel sample was then incubated in TFBA solution (0.004734 mmol/20 μL) for 30 min, then washed with THF, and dried.

#### 2.2.5. Functionalization of cryogels with fluorescein amine

The amount of fluorescein amine was calculated according to the amount of isocyanate in the cryogel. C1 cryogel contains 0.00118 mmol isocyanate. Fluorescein amine, 10 times more than the isocyanate in the cryogel, was used in immobilization, and 0.0118 mmol fluorescein amine for 5 mg of C1 cryogel was prepared in 30 μL of THF. As a control gel, 5 mg of C1 cryogel was kept in a solution prepared in 0.0118 mmol (0.8825 mg) AP and 20 μL of THF for 30 min. At the end of this period, washing was done with THF and then 5 mg of C1, and 5 mg of C1-3AP cryogels were placed in the prepared fluorescein amine solution and left for 30 min. After that, the cryogels were washed several times with THF and dried in vacuo.

#### 2.2.6. Functionalization of cryogels with FITC-BSA

An FITC labeled BSA solution was prepared at a concentration of 1 mg/1 mL in phosphate saline buffer (PBS). Then 5 mg of C1, C2, and C3 gels and C1-AP gel as a control were separately incubated in 20 μL of BSA solution and left for 1 h. At the end of that time, the cryogels were washed with phosphate buffer and then water and dried in vacuo. The gels were visualized by fluorescence microscopy.

## 3. Results and discussion

**Figure 1:**
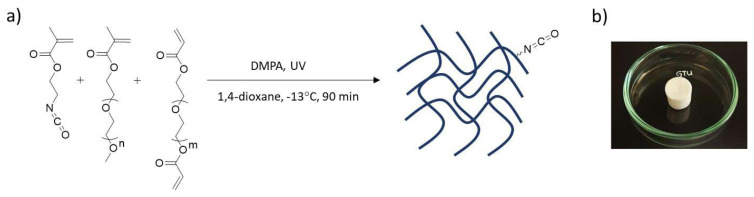
(a) Synthesis of cryogels via photopolymerization and (b) photograph of a cryogel sample (C1).

Reactive and hydrophilic cryogels containing side chain isocyanates were synthesized using PEGMEMA and ICEMA, with PEGDA as a crosslinker. The fabrication of cryogels was carried out by photopolymerization under UV light in the presence of DMPA as a photoinitiator (Figure 1). Since the isocyanate group is not very stable in water, the gel precursor was prepared in an organic solvent, namely 1,4-dioxane. First, homogeneous gel precursor was frozen at −70 °C, then placed in an antifreeze solution at −13 °C, and exposed to UV irradiation for 1.5 h. The resulting cryogels were subsequently washed with 1,4-dioxane and THF and dried in vacuo. They were obtained as opaque and soft materials in the shape of the reaction vessel in high yields. Cryogels C1–C3 were obtained in yields of 93.37 ± 0.78%, 83.07 ± 0.11%, and 85.15 ± 0.77%, respectively (Table). The yield of the gels was calculated as shown in Eq. (3).


(3) $$Yield\;(\%)=\frac{w\;(dry\;cryogel)}{w\;(total\;weight\;of\;monomers+crosslinkers)}$$

**Figure 2:**
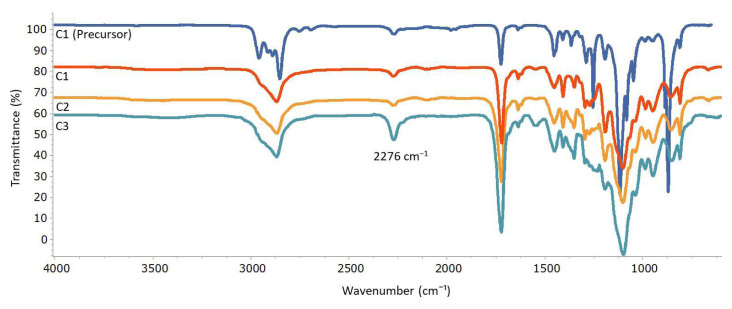
Comparison of FTIR spectra of before cryogelation of C1 (precursor) and cryogels prepared with different ICEMA content.

The presence of the reactive isocyanate group within the cryogels and its stability were confirmed through the prominent absorption peak at approximately 2276 cm^−1^, corresponding to the N═C═O stretching band, as observed in the analysis of both the gel precursor of C1 and the washed and dried C1 (Figure 2). As the mole ratio of ICEMA monomer in the precursor was increased from 10% to 40% (from C1 to C3), a consistent rise in the intensity of the N═C═O stretching bands was observed, as expected. This demonstrates that the quantity of isocyanate groups within the gels can be readily controlled by adjusting the ratio of monomer utilized in the gel precursors.

**Figure 3:**
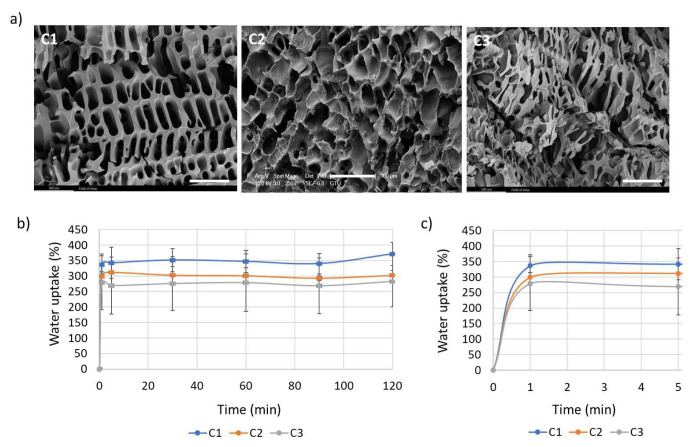
(a) SEM images of the cryogels (scale bars = 100 μm), (b) average pore size and pore size distribution of the cryogels, (c) water uptake graph of the cryogels (0–120 min), and (d) an expanded view of the water uptake graph for the first 5 min.

Morphological analysis of synthesized cryogels was performed by SEM. The cryogels had a highly porous morphology as seen in the SEM analysis (Figure 3a). As the freezing temperature decreases, pore alignment increases due to the freezing rate of the gel precursor increasing and ice crystals forming and growing in a certain direction [44]. Consistent with the literature, the cryogels were observed to have aligned pores due to the low temperature at which they were frozen [45]. The porous structure of the cryogels was investigated using SEM measurements. It was observed that the pore sizes of the cryogels were compatible with the monomer amounts during cryogelation. Consistent with the literature, it was observed that as the PEGMEMA ratio decreased, the pore diameter also decreased [46]. The pore diameters of C1, C2, and C3 cryogels were calculated to be 48 ± 15 μm, 39.9 ± 14.5 μm, and 34.5 ± 13.8 μm, respectively (Figure 3b). The synthesized cryogels exhibited swelling in water due to the hydrophilic PEGMEMA. Thanks to the use of hydrophilic PEGMEMA and the high porosity of the matrix, the synthesized gels swelled rapidly within 1 min. Additionally, as expected, as the PEGMEMA ratio increased, the percent water uptake of the cryogels also increased [47] (Figure 3c). It was observed that C1 gel had the highest swelling capacity and had swelled by 371.33% by the end of 2 h.

The deswelling–reswelling cycle of the cryogels is shown in Figure S1. The reswelling of the gels when placed in water after acetone shows that it has a stable pore structure that does not collapse during deswelling and allows water diffusion [48].

**Figure 4:**
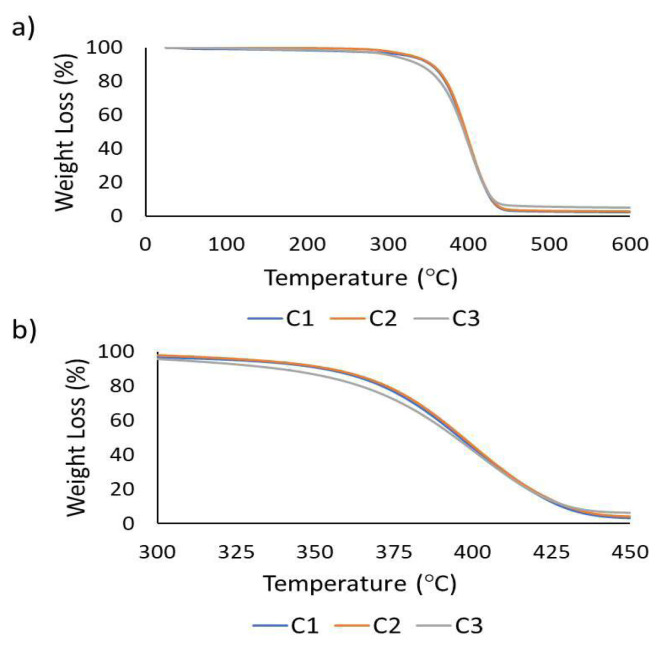
TGA of cryogels (a) 20–160 °C and (b) 300–450 °C.

The thermal stabilities of the synthesized cryogels were assessed using TGA (Figure 4). Dry cryogel samples were subjected to heating at a rate of 10 °C/min, ranging from 0 °C to 600 °C. Thermograms of the cryogels exhibited a single wave pattern. It is noteworthy that the cryogels exhibited stability up to 318 °C, irrespective of their chemical composition. However, variations were observed depending on the concentration of ICEMA/PEGMEMA within the cryogel. The decomposition of C1 cryogel commenced at 318.27 °C, while C2 cryogel showed decomposition at 356.67 °C, and C3 cryogel, containing the highest concentration of isocyanate groups, displayed decomposition at 360.49 °C. Furthermore, the temperature corresponding to the maximum degradation rate (Tpeak, the first derivative peak temperature) was determined as 386.99 °C for C1, 404.50 °C for C2 (which had an increased amount of isocyanate), and 407.29 °C for C3 (containing the most isocyanate groups). It was observed that thermal stability slightly decreases as the PEGMEMA ratio increases. This behavior might be attributed to the higher mobility of the system due to the length of the PEGMEMA aliphatic chains [44].

**Figure 5:**
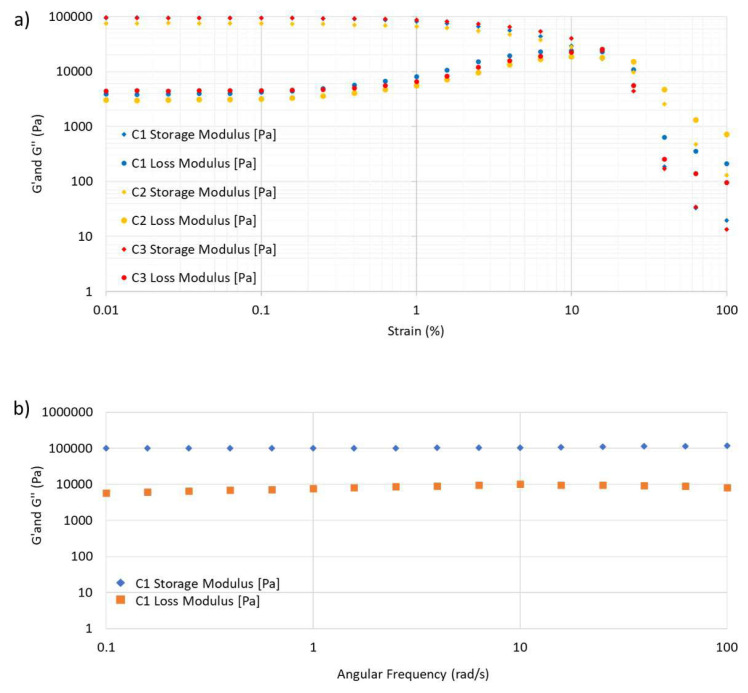
(a) Strain sweep test of the hydrogel and (b) frequency sweep test of C1.

When 0.01%–100% strain was applied to the cryogels, linear viscoelastic behavior was observed up to 1% strain under 10 rad/s frequency (Figure 5a) and cryogels that were prepared with different ratios of monomer showed similar properties. The rheological analyses indicate that the synthesized materials have a storage modulus (G′) greater than the loss modulus (G″), demonstrating their elastic property since high storage modulus (G′) implies a greater ability of the material to store deformation energy in an elastic manner. Consistent with the literature, it has been observed that the storage modulus is higher at low strains, while the storage modulus decreases and the loss modulus increases as the strain increases [49]. The transition point of the material (the point where G″ equals G′) is the gel breaking point [50]. The response of C1 to scaled frequency was measured by the frequency sweep test from 0.1 to 100 rad/s with a constant strain of 1% (Figure 5b). In the frequency sweep test, the storage modulus is higher than the loss modulus, indicating that the gel has natural high elasticity [51,52].

**Figure 6:**
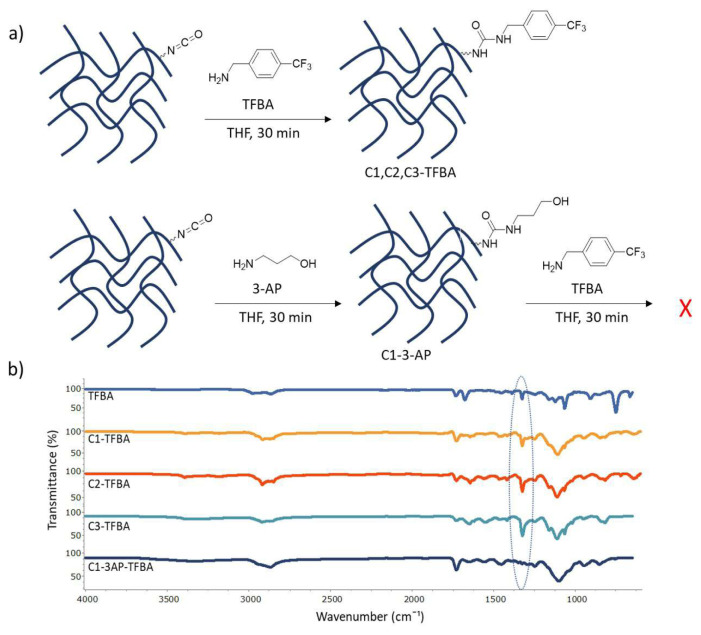
(a) Conjugation of TFBA and 3-AP to cryogels and (b) their FTIR spectra.

To demonstrate that amine-containing molecules were immobilized to cryogels, a primary amine containing TFBA was conjugated in THF for 30 min and characterized by FTIR analysis. The FTIR spectrum of the cryogels is shown in Figure 6. When TFBA is conjugated to C1–C3 cryogels, the disappearance of the isocyanate peak at ~2270 cm^−1^ after binding and the formation of a new peak at ~1324 cm^−1^ due to C–F stretching indicate that the binding has taken place successfully. It was shown that TFBA did not bind to the C1 gel used as a control after AP (Figure S2), and the intensity of the peaks resulting from CF_3_ stretching vibration increased as the ratio of isocyanate units in the cryogels increased.

**Figure 7:**
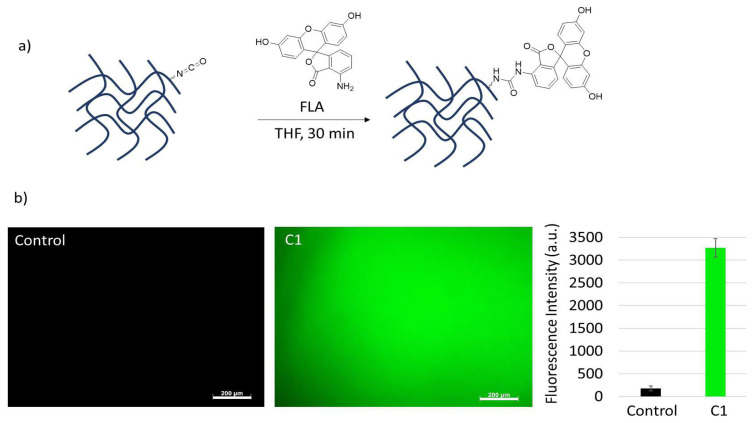
(a) General scheme of conjugation of FLA to cryogels and (b) their fluorescence microscope image and fluorescence intensities.

To visualize the functionalization of cryogels with amine-containing molecules, an amine-containing fluorescent dye, FLA, was utilized to label the cryogels. To achieve this, both the control gel, which was modified with AP to block isocyanates, and C1 cryogels were immersed in a solution containing FLA. Subsequently, the modified cryogels were thoroughly washed to eliminate any excess and/or nonconjugated dye. The surfaces were then examined using a fluorescence microscope. The results demonstrated successful conjugation of the fluorescent dye to the C1 cryogel through the amine–isocyanate reaction, aligning with our expectations. Conversely, as anticipated, no binding was observed in the control gel (Figure 7).

**Figure 8:**
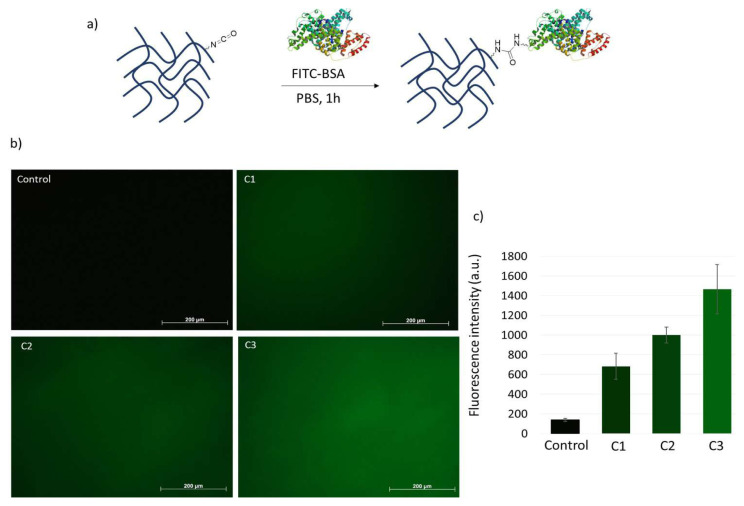
(a) Scheme of conjugation of FITC-BSA to cryogels, (b) fluorescence microscope images of FITC-BSA conjugated cryogels, and (c) relative fluorescence intensities.

To demonstrate the direct attachment of amine-containing biomolecules to the synthesized cryogels, we investigated the immobilization of FITC-BSA. The cryogels were immersed in a FITC-BSA solution for 1 h. Subsequently, the modified cryogels were thoroughly washed to remove any unbound BSA, and the surfaces were examined using a fluorescence microscope. As anticipated, the fluorescence intensity increased in accordance with the ratio of reactive groups within the cryogels (Figure 8). Notably, in the control gel obtained by coupling AP to the C1 gel, no significant fluorescence was detected following treatment with the FITC-BSA solution.

## 4. Conclusion

We present the first example of reactive isocyanate-containing cryogels synthesized through photopolymerization. Cryogelation was conducted in an organic solvent to prevent the decomposition of isocyanate groups during the process. The successful incorporation of the isocyanate monomer into the cryogelation process and its stability were confirmed through FTIR analysis. The resulting cryogels exhibited properties of being opaque, highly porous materials. Notably, we demonstrated the rapid and catalyst-free functionalization of these cryogels with amine-containing small molecules in an organic solvent, as well as with a fluorescence-labeled protein in an aqueous solution. The controlled immobilization of the protein was verified using a fluorescence microscope, as the fluorescein intensity increased proportionally with the ratio of isocyanate bearing monomer in the precursor. We think that these highly porous and easily functionalizable materials have great potential for applications in various (bio)immobilization-based research areas.

## Supplementary Data

Figure S1Deswelling–reswelling kinetics of the cryogels.Number of runs = 1st ( 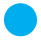), 2nd ( 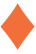), 3rd ( 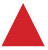), A = Acetone W = water

Figure S2 FTIR spectra of C1, 3-AP, and 3-AP immobilized C1 cryogel.

## Figures and Tables

**Table:** Cryogels fabricated via photocrosslinking.

Cryogel	ICEMA:PEGMEMA	ICEMA (mmol)	PEGMEMA (mmol)	PEGDA (mmol)	Yield%
C1	10:40	0.13	0.52	0.65	93.37 ± 0.78
C2	25:25	0.325	0.325	0.65	83.07 ± 0.11
C3	40:10	0.52	0.13	0.65	85.15 ± 0.77
