# Supplementary figures and images for: Synthesis of isocyanate containing cryogels for fast (bio)molecular immobilization
Source: Turk J Chem. 2024 Oct 8;48(5):770–9. doi: 10.55730/1300-0527.3696 (PMC11539910; doi:10.55730/1300-0527.3696)

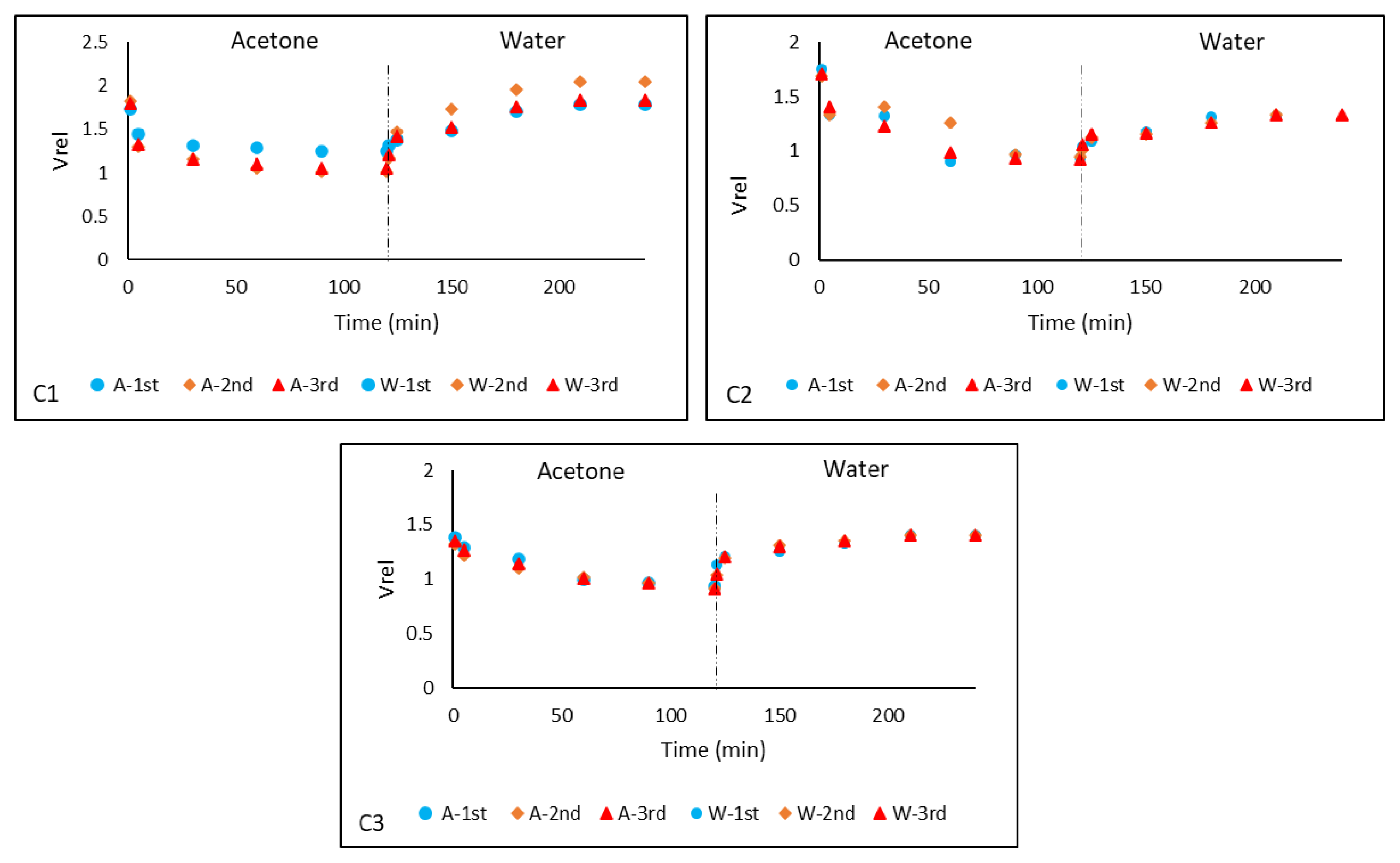

Supplement: Figure S1 — Deswelling–reswelling kinetics of the cryogels. Number of runs = 1st ( ), 2nd ( ), 3rd ( ), A = Acetone W = water [file tjc-48-05-770s1.tif]

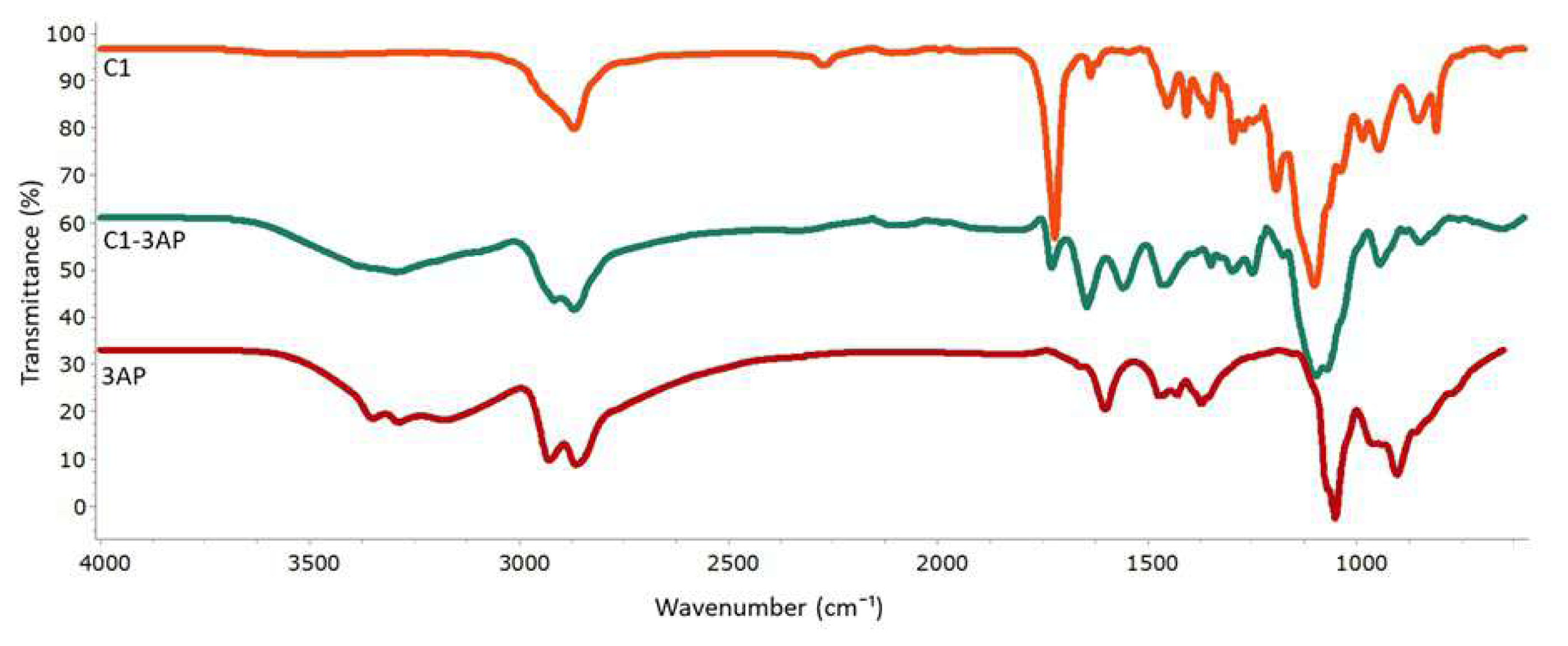

Supplement: Figure S2 — FTIR spectra of C1, 3-AP, and 3-AP immobilized C1 cryogel. [file tjc-48-05-770s2.tif]
